# Circulating myeloid-derived suppressor cells may be a useful biomarker in the follow-up of unvaccinated COVID-19 patients after hospitalization

**DOI:** 10.3389/fimmu.2023.1266659

**Published:** 2023-11-14

**Authors:** Carlos Jiménez-Cortegana, Elena Salamanca, Natalia Palazón-Carrión, Flora Sánchez-Jiménez, Antonio Pérez-Pérez, Teresa Vilariño-García, Sandra Fuentes, Salomón Martín, Marta Jiménez, Raquel Galván, Carmen Rodríguez-Chacón, Catalina Sánchez-Mora, Elisa Moreno-Mellado, Belén Gutiérrez-Gutiérrez, Nerissa Álvarez, Alberto Sosa, José Garnacho-Montero, Luis de la Cruz-Merino, Jesús Rodríguez-Baño, Víctor Sánchez-Margalet

**Affiliations:** ^1^ Department of Medical Biochemistry and Molecular Biology, School of Medicine, Virgen Macarena University Hospital, University of Seville, Seville, Spain; ^2^ Department of Laboratory Medicine, Virgen Macarena University Hospital, Seville, Spain; ^3^ Infectious Diseases and, Microbiology and Preventive Medicine Unit, Virgen Macarena University Hospital/Departments of Medicine and Microbiology, University of Seville/Biomedicine Institute of Seville (IBiS), Seville, Spain; ^4^ CIBERINFEC, Instituto de Salud Carlos III, Madrid, Spain; ^5^ Clinical Oncology Service, Virgen Macarena University Hospital, University of Seville, Seville, Spain; ^6^ Intensive Care Unit, Virgen Macarena University Hospital, Seville, Spain

**Keywords:** SARS-CoV-2, COVID-19, MDSCs, T lymphocytes, biomarker

## Abstract

SARS-CoV-2 infection is the cause of the disease named COVID-19, a major public health challenge worldwide. Differences in the severity, complications and outcomes of the COVID-19 are intriguing and, patients with similar baseline clinical conditions may have very different evolution. Myeloid-derived suppressor cells (MDSCs) have been previously found to be recruited by the SARS-CoV-2 infection and may be a marker of clinical evolution in these patients. We have studied 90 consecutive patients admitted in the hospital before the vaccination program started in the general population, to measure MDSCs and lymphocyte subpopulations at admission and one week after to assess the possible association with unfavorable outcomes (dead or Intensive Care Unit admission). We analyzed MDSCs and lymphocyte subpopulations by flow cytometry. In the 72 patients discharged from the hospital, there were significant decreases in the monocytic and total MDSC populations measured in peripheral blood after one week but, most importantly, the number of MDSCs (total and both monocytic and granulocytic subsets) were much higher in the 18 patients with unfavorable outcome. In conclusion, the number of circulating MDSCs may be a good marker of evolution in the follow-up of unvaccinated patients admitted in the hospital with the diagnosis of COVID-19.

## Introduction

SARS-CoV-2 infection can lead to a wide range of symptoms and clinical manifestations, ranging from mild cold-like symptoms to fatal outcomes. Patients admitted to the hospital with COVID-19 may experience a spectrum of disease severity, with some individuals being discharged within a week due to mild disease, while others may develop severe bilateral pneumonia requiring oxygen and respiratory support in the Intensive Care Unit (ICU), and sadly, some may succumb to the infection. The mortality rate of COVID-19 has been estimated to be between 1% and 2% of infected patients, depending on factors such as age and co-morbidities (1, 2).

Myeloid-derived suppressor cells (MDSCs) constitute a heterogeneous group of immature immune cells originating from the myeloid lineage. This cell population is recruited and expanded in the inflammation sites *through* C-C motif chemokine ligand 2 (CCL2)/C-C motif chemokine receptor 2 (CCR2), CCL3/CCR5 or CXC motif chemokine ligand 13 (CXCL13)/CXC motif receptor 5 (CXCR5) pathways, and granulocyte-macrophage colony-stimulating factor (GM-CSF), interleukin (IL-6), or prostaglandin E2 (PGE2). MDSCs not only exhibit immunosuppressive functions on T-cell responses, but also participate in the suppression of dendritic cell (DC)-, B- and NK-mediated immune responses and have a role in the development and expansion of other suppressor cells such as regulatory T cells (Tregs) or M2-macrophages (3, 4).

Under normal conditions, these cells differentiate into mature macrophages, DCs, or granulocytes. However, under pathological conditions like cancer or viral and bacterial infections, this process may shift towards a pathological and immunosuppressive phenotype (5–9). Mouse MDSCs are characterized as Gr1^+^CD11b^+^ cells with expression of Ly6C^high^Ly6G^-^ and Ly6C^low^Ly6G^+^, which are called monocytic (M-) and granulocytic (G-)MDSCs, respectively. In contrast, human MDSCs remain less defined. They are CD11b^+^HLA-DR^low/-^ cells that also express CD33+ and CD14^+^CD15^-^ (M-MDSCs) or CD14^-^CD15^+^ (G-MDSCs). Of note, G-MDSCs can also express high levels of CD66b (10, 11).

In previous research, we observed that patients with mild COVID-19 exhibited increased levels of blood M-MDSCs, along with lymphopenia, an increased number of exhausted T cells, and a lower number of activated T cells, compared to healthy donors. Furthermore, blood M-MDSCs showed a negative correlation with activated T cells (12). In a recent study, we also found that peripheral blood G-MDSCs could serve as a marker for mortality in ICU-admitted patients with COVID-19 (13). Other studies have also suggested that MDSCs might influence both disease severity and mortality (14, 15). However, there is ongoing debate about which MDSC subtype (M-MDSCs or G-MDSCs) may predict COVID-19 severity (15, 16). Additionally, MDSCs have been proposed not only as potential biomarkers but also as therapeutic targets in COVID-19 (17).

MDSCs are known to inhibit T lymphocytes, leading to their inactivation, exhaustion, and apoptosis (18). In the context of COVID-19, T lymphocytes from patients have been found to exhibit increased expression of inhibitory markers, such as PD-1 or CTLA-4, which contribute to an ineffective immune response (19, 20). Moreover, exhausted CD4+ T cells have been associated with poor outcomes in COVID-19 (21). Consistent with these findings, we have observed a positive correlation between the numbers of MDSCs and exhausted T cells in severe COVID-19 patients admitted to the ICU (13). Conversely, we have found a lower number of activated T lymphocytes (CD3+ expressing OX-40) in COVID-19 patients (12, 13). Similar results have been reported for CD3+ expressing HLA-DR (22). Furthermore, we noted a negative correlation between MDSCs and activated T lymphocytes in both mild and severe COVID-19 patients (12, 13), suggesting that the increase in MDSCs might contribute to the prevention of T-cell activation.

Given these observations, we now seek to investigate whether MDSCs could serve as a valuable marker for the follow-up of COVID-19 patients, predicting their disease evolution from admission to discharge in cases of mild disease or ICU admission or exitus in cases of severe disease. To achieve this, we conducted a study on 90 consecutive patients admitted to the hospital during the second and third waves of COVID-19 before the initiation of the vaccination program in the general population.

## Materials and methods

### Study design

This is a prospective, observational cohort study that included adult patients (age ≥ 18 years) with COVID-19 admitted to the Virgen Macarena University Hospital in Seville, Spain, from January 2021 to March 2021. We excluded patients with previous immunosuppression, such as those with solid organ or hematologic transplantations, hematologic malignancies, or those taking immunosuppressants before hospital admission, as well as pregnant women.

The clinicopathological characteristics of the enrolled patients are presented in **Table 1**.

**Table 1:** Clinico-pathological data of the COVID-19 patients included in this study.

Characteristics	N (%)
Patients	90 (100)
Age (years) *	61 (56-65)
Female sex	35 (38.89)
Comorbidities Chronic heart failure Cancer Chronic kidney disease Liver cirrhosis Diabetes mellitus	3 (3.33) 9 (10.00) 7 (7.78) 1 (1.11) 12 (13.33)
ICU hospitalization	12 (13.33)
Mechanical ventilation	22 (24.44)

*Data are shown as median and 95% confidence interval.

### Patients

We studied the immunological characteristics of peripheral blood cells from 90 COVID-19 patients admitted at the Virgen Macarena University Hospital (Seville, Spain) after being tested positive as previously described (12, 13). Blood was obtained at admission and one week later, using samples sent to the hospital laboratory for routinary tests. Patients were divided into two groups, those with worst outcome (6 patients who died after one week of hospitalization and 12 patients admitted at ICU) and 72 patients who had a good evolution and were discharged from the hospital after the same follow-up time. The study was approved by the Institutional Review Board (12, 13).

### Flow cytometry analysis in whole blood samples

Cell populations were assessed using the FACSCanto II flow cytometry system (Becton Dickinson, Franklin Lakes, NJ, USA) from EDTA-K3 tubes. MDSCs and lymphoid populations, including T, B, NK, CD4+, and CD8+ T cells, as well as Tregs, PD-1+OX40− T, and OX40+PD-1− T cells, were gated as previously described (12, 13). HLA-DR-expressing T cells were gated as illustrated in **Supplementary Figure 1**: CD3+CD8+HLA-DR+ and CD3+CD8-HLA-DR+. The latter population was considered as part of the CD4 compartment; therefore, we referred to it as CD4+HLA-DR+ T cells. The absolute count of these T-cell subsets was calculated by multiplying the percentage of CD4+HLA-DR+ and CD8+HLA-DR+ by the absolute count of CD4+ and CD8+ T cells, respectively. The absolute count of total HLA-DR+ T cells was calculated by summing up the CD3+CD4+HLA-DR+ cell counts and CD3+CD8+HLA-DR+ cell counts. Additionally, total lymphocyte, monocyte, and granulocyte counts were obtained from hematologic counts (Sysmex CS-1000). **Table 2** displays the cell counts of granulocytes, lymphocytes, T cells, CD4+ T cells, CD8+ T cells, B cells, and NK cells.

**Table 2:** Granulocyte and lymphoid population levels in COVID-19 patients.

Cell population	Determinations	Discharge from ES	Exitus from ES/ICU admission	P value (Between groups)	P value (Between determinations)	
Granulocytes	Hospitalization	6290 (5790-7250)	5885 (4260-7460)	0.6945	Discharge	*<0.0001*
	One week later	7375 (6270-9555)	10220 (7460-10810)	*0.0064*	Exitus/ICU	*0.0012*
Total lymphocytes	Hospitalization	1035 (920-1290)	670 (500-950)	*0.0008*	Discharge	*<0.0001*
	One week later	1265 (1040-1520)	560 (330-1270)	*0.0005*	Exitus/ICU	0.5300
T cells	Hospitalization	625 (488-710)	425 (265-623)	*0.0124*	Discharge	*<0.0001*
	One week later	794 (653-1044)	287 (220-790)	*0.0003*	Exitus/ICU	0.9460
B cells	Hospitalization	139 (124-166)	95 (45-149)	*0.0084*	Discharge	*<0.0001*
	One week later	198 (156-280)	119 (45-193)	*0.0132*	Exitus/ICU	0.0681
NK cells	Hospitalization	206 (166-249)	163 (91-216)	*0.0231*	Discharge	*0.0002*
	One week later	179 (147-209)	85 (29-140)	*<0.0001*	Exitus/ICU	*0.0005*
CD4+ T cells	Hospitalization	388 (298-447)	212 (152-372)	*0.0094*	Discharge	*<0.0001*
	One week later	493 (400-639)	193 (138-552)	*0.0014*	Exitus/ICU	0.2734
CD8+ T cells	Hospitalization	188 (147-223)	127 (81-228)	*0.0455*	Discharge	*0.0022*
	One week later	221 (185-328)	96 (67-169)	*0.0001*	Exitus/ICU	*0.0398*

Cell concentrations are represented as median and 95% confidence intervals of cells per microliter. Statistically significant differences (p<0.05) appear in italics.

### Monoclonal antibodies

All antibodies were acquired from Becton Dickinson Immunocytometry Systems (San Jose, CA, USA) and employed at the concentrations recommended by the manufacturer (12, 13).

*MDSCs*: PerCP-Cy5.5 Mouse anti-human CD 45 (ref no. 564105), APC-Cy7 rat anti-CD11b (ref no. 557657), PE mouse anti-Human CD 33 (ref no. 555450), PE-Cy7 mouse anti-human HLA-DR (ref no. 560651), FITC mouse anti-human CD 14 (ref no. 555397) and APC mouse anti-human CD 15 (ref no. 551376).
*Tregs*: Human Treg cocktail (ref no. 560249), which includes PerCP Mouse Anti-Human CD4, PE mouse anti-Human CD127, and FITC anti-human CD25.
*Activated OX40^+^PD-1^-^ T cells*: FITC Mouse anti-human OX-40 (CD134) (ref no. 555837), PerCP-Cy5.5 mouse anti-human CD8 (ref no. 565310), PE-Cy7 mouse anti-human CD4 (ref no. 557852), APC mouse anti-human PD-1 (CD279) (ref no. 558694), APC-H7 mouse anti-human CD3 (ref no. 560176).
*Activated HLA-DR^+^ T cells*: PE-Cy7 mouse anti-human HLA-DR (ref no. 560651), PerCP-Cy5.5 mouse anti-human CD8 (ref no. 565310), APC-H7 mouse anti-human CD3 (ref no. 560176).

### Data analysis

Statistical analysis was conducted using GraphPad Prism 8.0.2 (GraphPad Software, San Diego, CA, USA), and graphs were generated accordingly. Continuous variables were presented as the median and 95% confidence intervals, while qualitative variables were expressed as absolute numbers and percentages. The normal distribution of analyzed variables was assessed through a histogram, box plot, the Q-Q plot, and the Kolmogorov-Smirnov normality test. As the data did not follow a normal distribution, non-parametric tests were employed. The comparison of cell distributions between discharged, deceased, or ICU-admitted COVID-19 patients was performed using the Mann-Whitney U test. Additionally, the Wilcoxon test was utilized to compare cell distributions within each group of patients. Bivariate correlations among cell populations were determined using Spearman’s coefficient. Statistically significant differences were considered for p-values ≤ 0.05.

## Results

### Clinico-pathological characteristics of COVID-19 patients

Ninety patients diagnosed with COVID-19 and hospitalized at the Virgen Macarena University Hospital (Seville, Spain) were included in the study. **Table 1** displays their clinicopathological baseline characteristics.

The median age of the patients was 61 years old (95% CI, 56-65). Among them, 61.96% were male. Among the patients, 18 individuals experienced an unfavorable outcome, with 8 deaths and 10 ICU admissions due to either in-hospital mortality or post-hospitalization ICU care. The remaining 72 patients were successfully discharged without requiring ICU admission.

**Table 2** presents the cell counts of granulocytes, lymphocytes, T cells, CD4+ T cells, CD8+ T cells, B cells, and NK cells from the patients.

### Blood MDSC levels determined outcomes of hospitalized COVID-19 patients after the one-week follow-up

**Figure 1:**
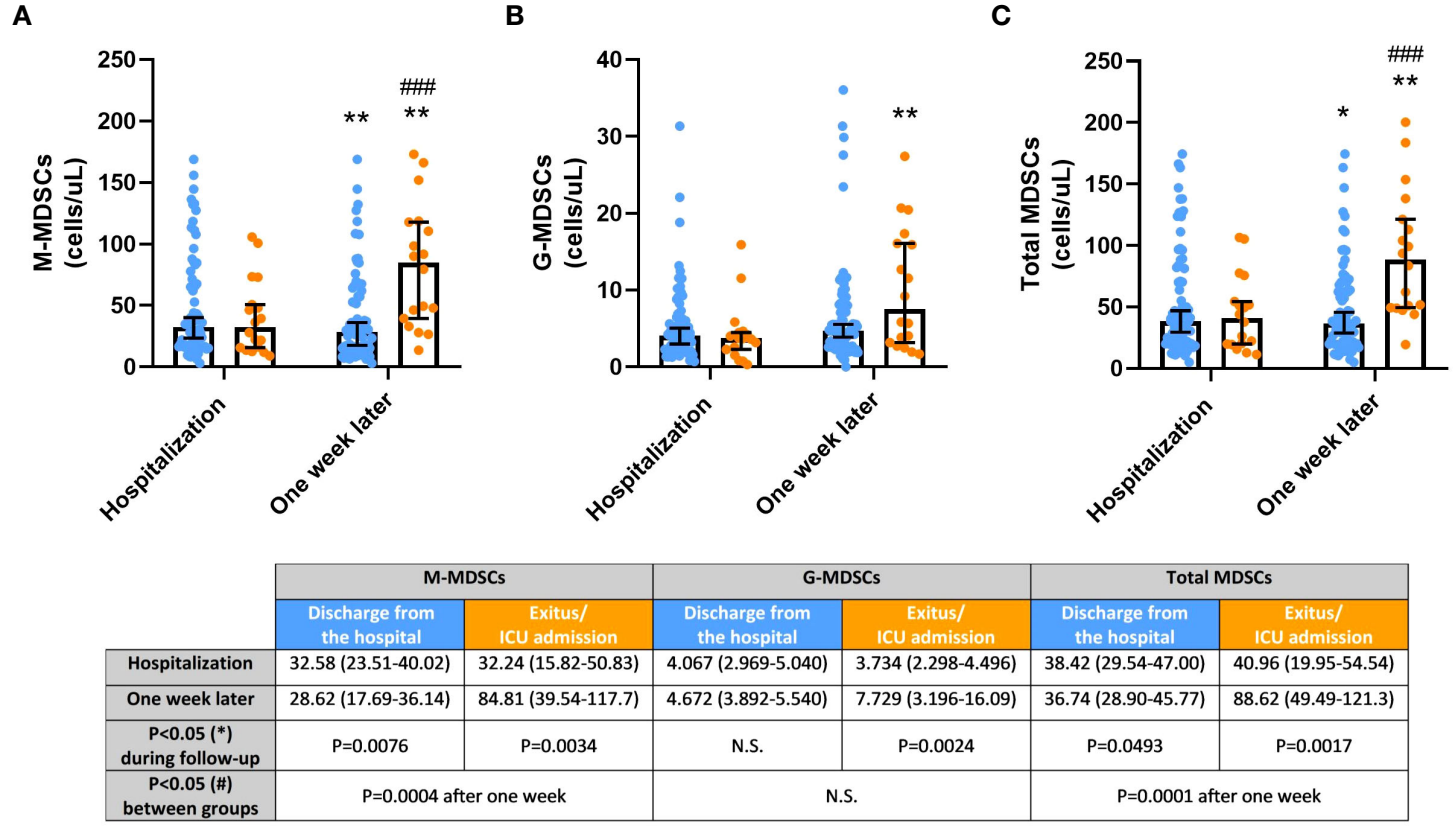
**(A)** Monocytic myeloid-derived suppressor cells (MDSCs); **(B)** Granulocytic MDSCs; **(C)** Total MDSCs, in COVID-19 patients discharged from the hospital (blue) and died after hospitalization or admitted at Intensive Care Unit (ICU; orange) during the one-week follow-up. Cell concentrations are represented as median and 95% confidence intervals (CI) of cells per microliter. *p ≤ 0.05 and **p ≤ 0.01, comparing determinations at hospitalization *vs*. one week later in every group of patients; ^###^p ≤ 0.001 comparing opposite groups in every determination. N.S., No significant.

The follow-up of circulating MDSCs from COVID-19 patients after hospitalization is shown in **Figures 1A–C**. MDSC levels (both M-MDSCs and G-MDSCs, as well as total MDSCs) were similar in all patients at admission in the Hospital. However, individuals with a unfavorable outcome experienced a significant increment of MDSC levels (M-MDSCs, p=0.0034; G-MDSCs, p=0.0024; and total MDSCs, p=0.0017) one week later. COVID-19 patients who were discharged from the hospital experienced a significant decrease of M-MDSCs (p=0.0076) and total MDSCs (p=0.0493), whereas G-MDSC levels remained constant. Interestingly, after the follow-up time, both M-MDSC and total MDSC levels were remarkably higher in the group of patients with worst outcome compared to those who were discharged from the hospital (p=0.0004 and p=0.0001, respectively).

### Blood Treg levels after hospitalization determined clinical evolution of COVID-19 patients during the one-week follow-up

The proportion of circulating Tregs displayed a remarkable difference between both groups of patients after one week of hospitalization, as depicted in **Supplementary Figure 2**. Patients with an unfavorable outcome exhibited constant, yet overall low levels of Tregs, whereas patients with a better outcome experienced a significant increase in Tregs one week after hospitalization (p<0.0001). When comparing the two groups, blood Treg levels were significantly lower in the group with an unfavorable outcome both at hospitalization (p=0.0289) and one week later (p=0.0002) compared to the discharged patients.

### OX40+PD-1- expression may activate cytotoxic T cells of COVID-19 patients discharged from the hospital

**Figure 2:**
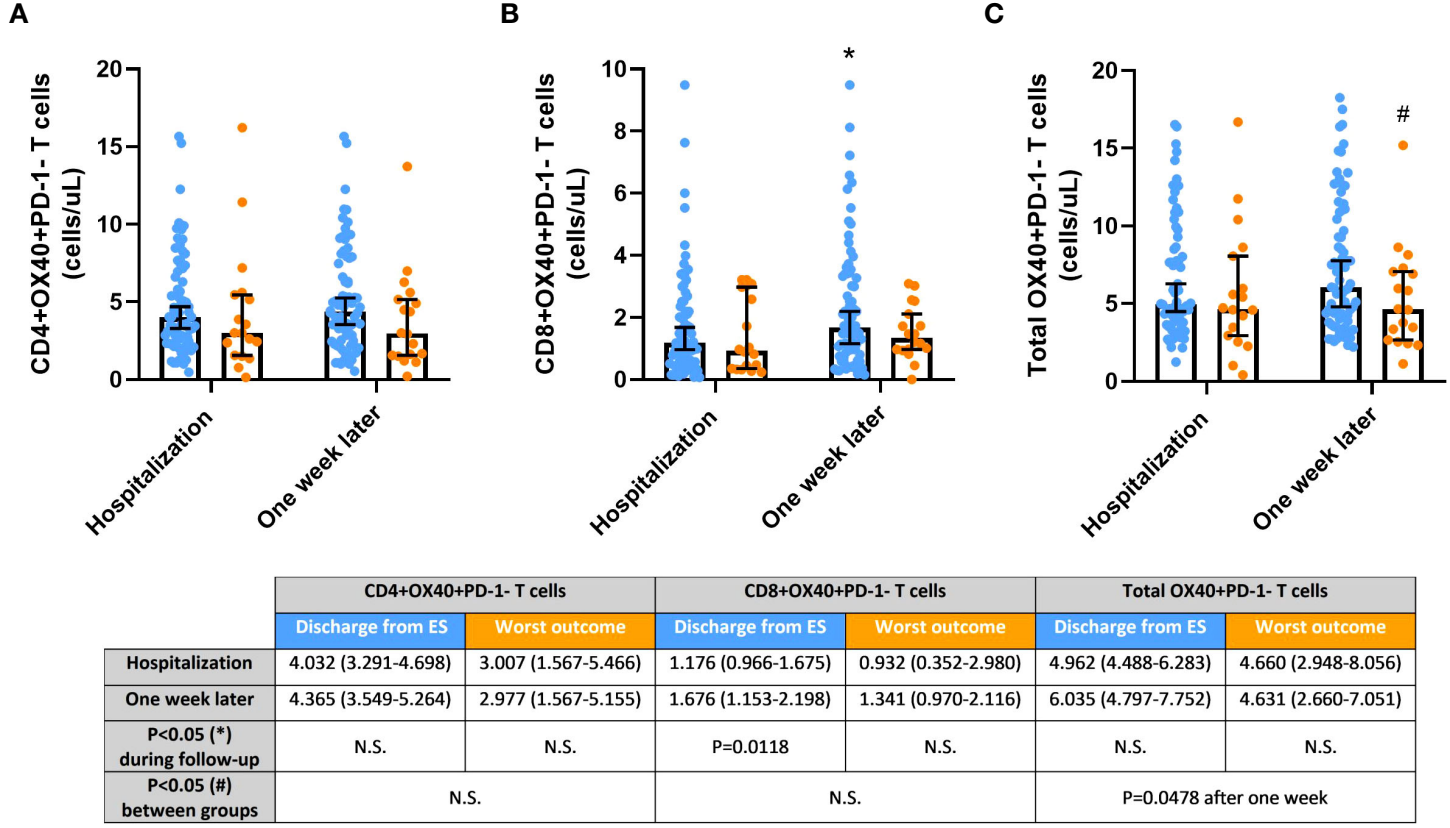
Activated OX40+PD-1- T cells, **(A)** CD4+; **(B)** CD8+; and **(C)** Total, in COVID-19 patients discharged from the hospital (blue) and passed away after hospitalization or admitted at Intensive Care Unit (ICU; orange) during the one-week follow-up. Cell concentrations are represented as median and 95% confidence intervals of cells per microliter. *p≤0.05, comparing determinations at hospitalization vs. one week later in every group of patients; ^#^p ≤ 0.05, comparing opposite groups in every determination. N.S., No significant.

**Figures 2A–C** displays the evaluation of activated (OX40+PD-1-) T cells. The levels of activated CD4+ T cells were slightly higher in patients with a better outcome after hospitalization and one week later. Conversely, activated CD8+ T cells showed a significant increase during the follow-up in this patient cohort (p=0.0027), suggesting an intriguing OX40-mediated cytotoxic T-cell activation to facilitate immune responses against the SARS-COV-2 virus. Consequently, differences in the levels of total activated T cells between the groups were evident after one week of hospital stay (p=0.0496).

### Activated HLA-DR-expressing T cells were significantly increased in blood of COVID-19 patients discharged after the one-week hospitalization

**Figure 3:**
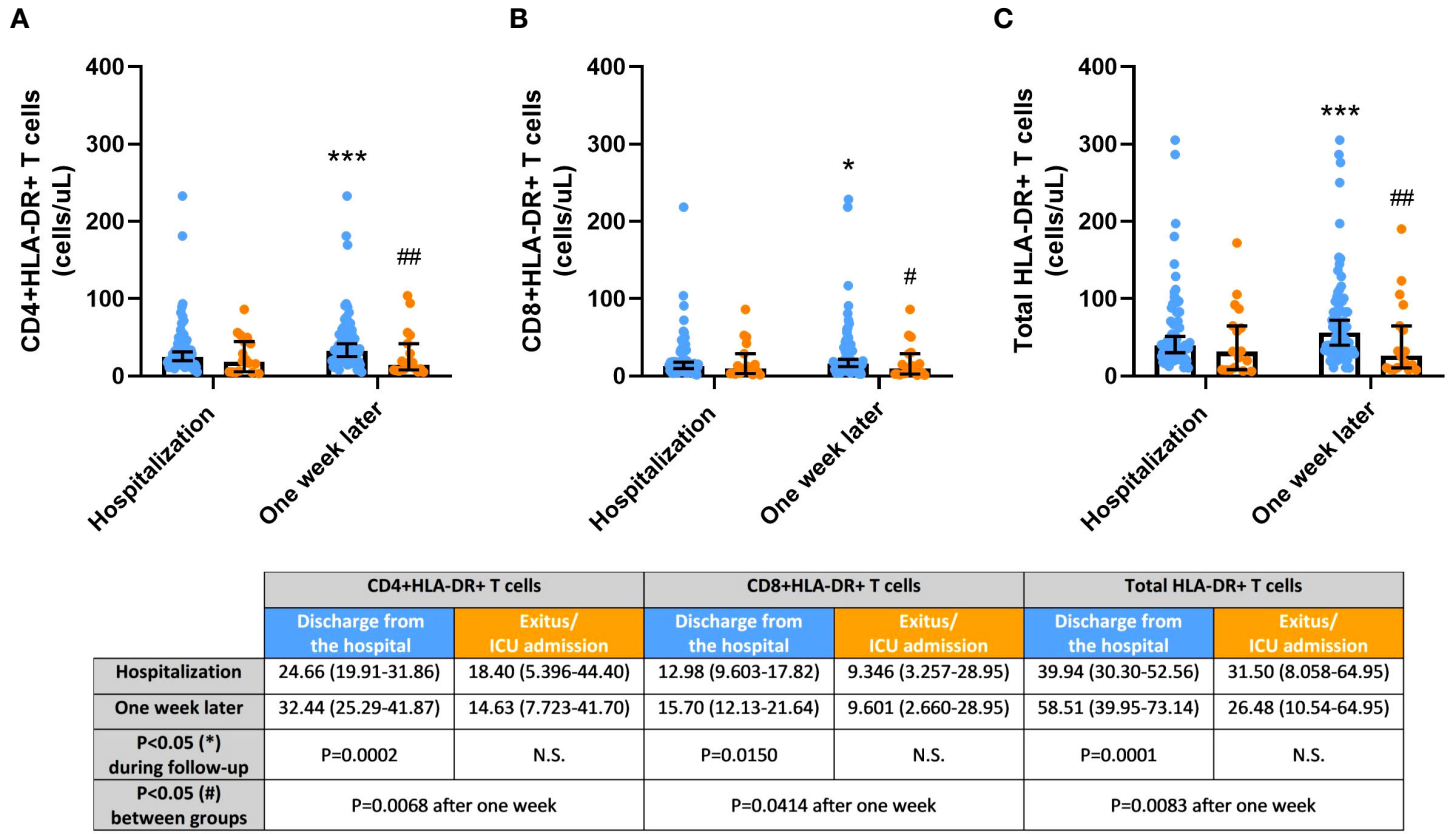
Activated HLA-DR+ T cells, **(A)** CD4+; **(B)** CD8+; and **(C)** Total, in COVID-19 patients discharged from the hospital (blue) and passed away after hospitalization or admitted at Intensive Care Unit (ICU; orange) during the one-week follow-up. Cell concentrations are represented as median and 95% confidence intervals of cells per microliter. *p ≤ 0.05 and ***p ≤ 0.001, comparing determinations at hospitalization *vs*. one week later in every group of patients; ^#^p ≤ 0.05 and ^##^p ≤ 0.01, comparing opposite groups in every determination. N.S., No significant.

HLA-DR expression on CD4+, CD8+, and total T cells was also analyzed in COVID-19 patients (**Figures 3A–C**). The concentration of these cell populations in the blood was similar in both groups after hospitalization but remained constant in the group with an unfavorable outcome. However, HLA-DR-expressing T cells significantly expanded in the blood of patients with a better outcome after the follow-up period (CD4+HLA-DR+ T cells, p=0.0002; CD8+HLA-DR+ T cells, p=0.0150; and total HLA-DR+ T cells, p=0.0001), leading to significant differences between groups after one week of hospitalization (CD4+HLA-DR+ T cells, p=0.0068; CD8+HLA-DR+ T cells, p=0.0414; and total HLA-DR+ T cells, p=0.0083).

### Activated OX40+PD-1- and HLA-DR+ T cells positively correlated each other and also with Tregs in COVID-19 patients

**Table 3** illustrates strong correlations observed between different cell populations. Tregs showed positive correlations with every population (CD4+, CD8+, and total) of both activated OX40+PD-1- and HLA-DR+ T cells (p<0.0001 in all cases). Additionally, all cell populations of activated T cells exhibited positive correlations with each other (p<0.0001 in all cases, except p=0.0003 for CD4+OX40+PD-1- T cells *vs*. CD8+HLA-DR+ T cells).

**Table 3:** Statistically significant (p<0.05) Spearman’s bivariate correlations between cell populations.

Cell populations	P value	Spearman’s coefficient
Tregs *vs*. CD4+HLA+ T cells	<0.0001	0.568
Tregs *vs*. CD8+HLA+ T cells	<0.0001	0.434
Tregs *vs*. Total HLA+ T cells	<0.0001	0.555
Tregs *vs*. CD4+OX40+PD-1- T cells	<0.0001	0.514
Tregs *vs*. CD8+OX40+PD-1- T cells	<0.0001	0.324
Tregs *vs*. Total OX40+PD-1- T cells	<0.0001	0.546
CD4+HLA+ T cells *vs*. CD4+OX40+PD-1- T cells	<0.0001	0.423
CD4+HLA+ T cells *vs*. CD8+OX40+PD-1- T cells	<0.0001	0.300
CD4+HLA+ T cells *vs*. Total OX40+PD-1- T cells	<0.0001	0.482
CD8+HLA+ T cells *vs*. CD4+OX40+PD-1- T cells	0.0003	0.275
CD8+HLA+ T cells *vs*. CD8+OX40+PD-1- T cells	<0.0001	0.470
CD8+HLA+ T cells *vs*. Total OX40+PD-1- T cells	<0.0001	0.414
Total HLA+ T cells *vs*. CD4+OX40+PD-1- T cells	<0.0001	0.387
Total HLA+ T cells *vs*. CD8+OX40+PD-1- T cells	<0.0001	0.389
Total HLA+ T cells *vs*. Total OX40+PD-1- T cells	<0.0001	0.482

## Discussion

COVID-19 exhibits a highly heterogeneous clinical evolution and outcomes, ranging from a mild cold-like illness to severe disease, influenced by various factors such as viral load, co-morbidities, and host immune response (23). However, the diverse responses of the innate immune system to the infection are not yet fully understood (2, 24). It is known that SARS-CoV-2 infection can lead to immune response suppression (25), partly due to the evasion of the innate immune system (26), resulting in significant lymphopenia (27, 28), which we previously detected in both mild and severe COVID-19 cases (12, 13). In this study, we found that patients with better outcomes (discharged from the hospital) had a higher proportion of lymphocytes at admission compared to those with an unfavorable outcome (deceased or admitted to the ICU). This suggests that low lymphocyte levels may predict severity or death in COVID-19 patients, as previously proposed (29, 30).

Suppression of T cell responses, as well as innate immune responses, is partly driven by the expansion of MDSCs, one of the major immunosuppressive mediators in the immune system (3). Consistent with this, we previously observed an increase in both circulating M-MDSCs in patients with mild COVID-19 (12) and G-MDSCs in patients with severe COVID-19 (13). Here, we demonstrate that MDSCs may serve as a useful biomarker for monitoring COVID-19 patients during one week of follow-up. Although all patients had similar clinical severity and levels of circulating MDSCs at admission, MDSC cell subsets significantly increased in the group of patients with worse outcomes after the follow-up period. Other studies have reported high levels of M-MDSCs in patients with acute COVID-19 (31), while others suggested G-MDSCs as the increased MDSC population in severe COVID-19 (14, 32).

Alternatively, MDSCs may also be a useful marker of immune suppression in COVID-19 patients with poor outcomes since Treg levels were low in peripheral blood, and even lower in those with a worse outcome after one week, whereas Tregs were increased in patients who were discharged from the hospital. The trend observed in the Tregs from peripheral blood of deceased/ICU-admitted patients suggests that Tregs may also be affected by the lymphopenic effects occurred in the SARS-COV-2 infection and may not mediate the immunosuppression driven by this viral disease (13). Also, Tregs has been previously found in a low percentage due to the proinflammatory cascade driven by IL-6, which could be diminished in recovered COVID-19 patients and may boost the levels of Tregs, as observed in the discharged COVID-19 patients after one week of follow-up. For these reasons, the application of Tregs has been proposed as a possible therapeutic approach to treat COVID-19 patients (33).

In addition, we analyzed T-cell activation by OX40 expression, as we did in previous studies with COVID-19 patients (12, 13). In this case, cytotoxic OX40+PD-1- T cells appear to be a promising biomarker for one-week follow-up since this cell population was significantly increased in discharged patients compared to those with poorer outcomes. To the best of our knowledge, previous reports regarding T-cell activation by OX40 have not been published by other research groups. However, we previously demonstrated the activation of both CD4+OX40+PD-1- and CD8+OX40+PD-1- T cells in patients discharged from the hospital, irrespective of the follow-up time (13). Here, we specifically checked the variations in the immune cell populations after one week of follow-up, and it appears that the activation of CD4+OX40+PD-1- T cells may take more time since no significant differences were found. The reason why only CD8+OX40+PD-1- T cells were increased in discharged patients after one week may be explained by the short period of stimulation that naïve CD8 T cells need to proliferate after activation (34). Also, the increase of CD8+OX40+PD-1- T cells may be sufficient for boosting T-cell responses in COVID-19 patients, as CD8 T cells play a critical role in mediating immune responses to acute viral infections in the lung (35).

Next, we analyzed activated HLA-DR-expressing T-cells. In previous studies involving COVID-19 patients, an overactivation of the immune system by HLA-DR+CD38+ T cells was found, even 12 months after infection (36), probably due to both the initial lymphopenia and the role these cells play in providing help to B lymphocytes (37–39). However, higher or lower expression of CD38 in HLA-DR+ T cells may promote different functions in co-activated T cells from severe COVID-19 patients (40). In this study, we analyzed the proportion of both circulating CD4+HLA-DR+ T and CD8+HLA-DR+ T cells (which may include variable CD38 expression). We observed that patients who were discharged from the hospital after one week of stay experienced a significant increase in every HLA-DR T cell population, suggesting that the activation of CD4 and CD8 T cells carried out by HLA-DR expression may be a promising biomarker for monitoring COVID-19 patients, at least during a short hospital stay. Importantly, we observed significant, positive correlations between every HLA-DR T cell population (CD4, CD8, and total) and every OX40+PD-1- T cell population (CD4, CD8, and total), highlighting the importance of T-cell activation biomarkers in monitoring COVID-19 patients. The evolution of this cell population (HLA-DR T cells without considering the expression of CD38) during the follow-up of COVID-19 patients has not been studied previously. However, HLA-DR T cells have been found increased in COVID-19 patients compared to healthy donors, especially the cytotoxic subset (41).

The inhibition of T cell activation may be mediated by both M-MDSC and G-MDSC (11). In fact, we have found lymphopenia and increased M-MDSC levels in mild COVID-19 (12), whereas higher G.MDSC levels were found in peripheral blood from severe COVI-19 patients (13). This expansion of G-MDSC could mediate at least in part the neutrophilia observed in severe COVID-19 patients, as previously suggested (42).

We have analyzed the immune profile of 90 unvaccinated, hospitalized COVID-19 patients, and we compared 72 individuals with good outcomes versus 18 individuals with worse outcomes. This imbalance in the sample size between groups makes us consider a limitation in the number of COVID-19 patients studied. Therefore, further studies in unvaccinated patients may be necessary to confirm the hypothesis that MDSCs may play a role in the evolution of COVID-19 patients admitted to the hospital. Additionally, considering that the vaccination program is already established and that some people have been reinfected, it could be interesting to study the immune profile of reinfected COVID-19 patients to consider MDSCs as a biomarker in the disease. Nevertheless, we have demonstrated that not only MDSCs may be considered as a biomarker in the follow-up of unvaccinated, hospitalized patients, and could be a plausible target for treatment in the infection with the SARS-CoV-2 virus, but also HLA-DR T cells may be a promising biomarker in the follow-up of these individuals.

## Data availability statement

The raw data supporting the conclusions of this article will be made available by the authors, without undue reservation.

## Ethics statement

The studies involving humans were approved by Virgen Macarena and Virgen del Rocio IRB. The studies were conducted in accordance with the local legislation and institutional requirements. The ethics committee/institutional review board waived the requirement of written informed consent for participation from the participants or the participants’ legal guardians/next of kin because of SARS-CoV-2 infection and to avoid contact.

## Author contributions

CJ: Investigation, Data curation, Formal Analysis, Methodology, Writing – original draft. ES: Investigation, Methodology, Writing – review & editing. NP: Investigation, Writing – review & editing. FS: Investigation, Writing – review & editing. AP: Investigation, Writing – review & editing. TV: Investigation, Writing – review & editing. SF: Investigation, Writing – review & editing. SM: Investigation, Writing – review & editing. MJ: Investigation, Writing – review & editing. RG: Investigation, Writing – review & editing. CR: Investigation, Writing – review & editing. CS: Investigation, Writing – review & editing. EM: Investigation, Writing – review & editing. BG: Investigation, Writing – review & editing. NÁ: Investigation, Writing – review & editing. AS: Investigation, Writing – review & editing. JG: Writing – review & editing, Conceptualization. LD: Conceptualization, Writing – review & editing. JR: Conceptualization, Writing – review & editing. VS: Conceptualization, Writing – review & editing, Funding acquisition, Investigation, Supervision.

## Supplementary material

Supplementary Figure 1 Gating of activated CD8-HLA-DR+ T cells and CD8+HLA-DR+ T cells. Since CD8-HLD-DR+ cells were considered as a population within the CD4 compartment, this subset may be named as CD4+HLA-DR+ T cells.

Supplementary Figure 2 Regulatory T cells in COVID-19 patients discharged from the hospital (blue) and passed away after hospitalization or admitted at Intensive Care Unit (ICU; orange) during the one-week follow-up. Cell concentrations are represented as median and 95% confidence intervals (CI) of cells per microliter. ****p ≤ 0.0001, comparing determinations at hospitalization *vs*. one week later in every group of patients. ^#^p ≤ 0.05, and ^###^p ≤ 0.001 comparing opposite groups in every determination.
