# Supplementary figures and images for: Circulating myeloid-derived suppressor cells may be a useful biomarker in the follow-up of unvaccinated COVID-19 patients after hospitalization
Source: Front Immunol. 2023 Nov 14;14:1266659. doi: 10.3389/fimmu.2023.1266659 (PMC10685891; doi:10.3389/fimmu.2023.1266659)

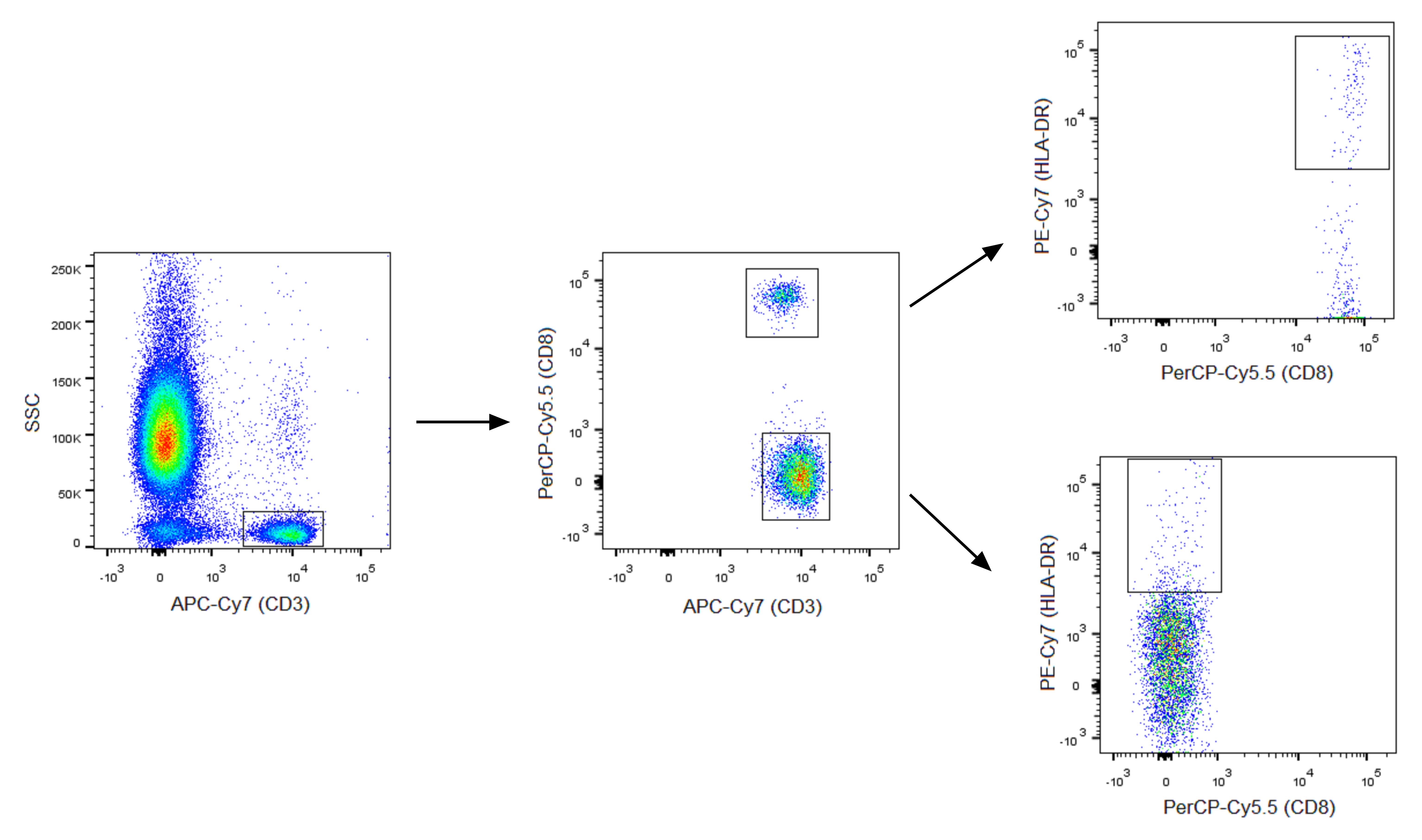

Supplement: Supplementary Figure 1 — Gating of activated CD8-HLA-DR+ T cells and CD8+HLA-DR+ T cells. Since CD8-HLD-DR+ cells were considered as a population within the CD4 compartment, this subset may be named as CD4+HLA-DR+ T cells. [file Image_1.jpeg]

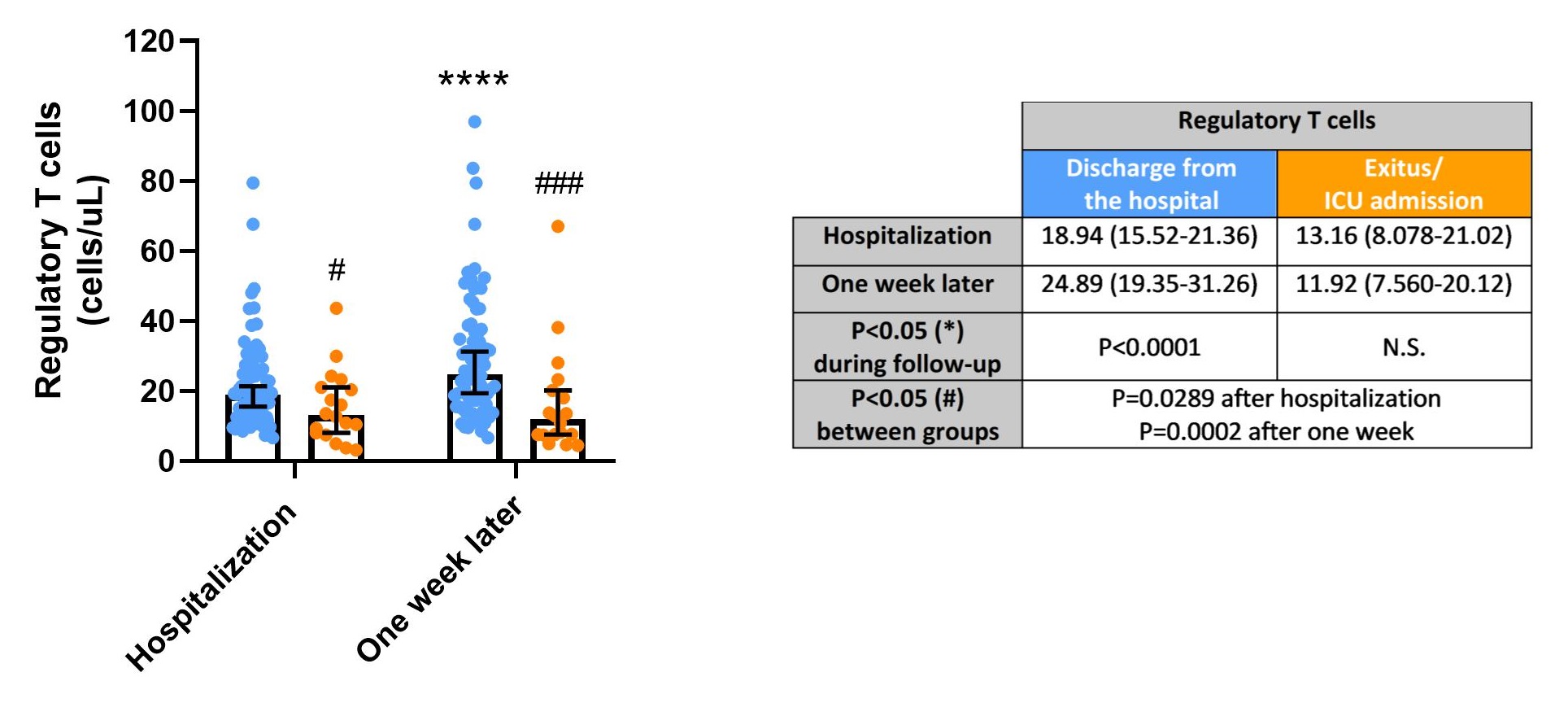

Supplement: Supplementary Figure 2 — Regulatory T cells in COVID-19 patients discharged from the hospital (blue) and passed away after hospitalization or admitted at Intensive Care Unit (ICU; orange) during the one-week follow-up. Cell concentrations are represented as median and 95% confidence intervals (CI) of cells per microliter. ****p ≤ 0.0001, comparing determinations at hospitalization vs. one week later in every group of patients. #p ≤ 0.05, and ###p ≤ 0.001 comparing opposite groups in every determination. [file Image_2.jpeg]
